# Development of a prototype modeling system to estimate the GHG mitigation potential of forest and wildfire management

**DOI:** 10.1016/j.mex.2022.101985

**Published:** 2022-12-23

**Authors:** C. Smyth, S.H. Xie, T. Zaborniak, M. Fellows, C. Phillips, W.A. Kurz

**Affiliations:** a Natural Resources Canada, Canadian Forest Service, 506 Burnside Road West, Victoria, BC V8Z 1M5, Canada; b University of British Columbia, 2424 Main Mall, Vancouver, BC V6T 1Z4, Canada; c Pacific Institute for Climate Solutions, PO Box 1700 Stn. CSC, Victoria, BC V8W 2Y2, Canada

**Keywords:** Wildfire reduction, Climate change mitigation, GCBM, Restoration, Prototype modeling system to estimate the GHG mitigation potential of forest and wildfire management

## Abstract

Having recently experienced the three worst wildfire seasons in British Columbia's history in 2017, 2018 and 2021, and anticipating more severe impacts in the future, a key Carbon (C) research priority is to develop reliable models to explore options and identify a portfolio of regionally differentiated solutions for wildfire and forest management. We contribute to this effort by developing a prototype integrated C modeling framework which includes future wildfires that respond to forest stand characteristics and wildfire history. Model validation evaluated net GHG emissions relative to a ‘do-nothing’ baseline for several management scenarios and included emissions from forest ecosystems, harvested wood products and substitution benefits from avoided fossil fuel burning and avoided emissions-intensive materials. Data improvements are needed to accurately quantify the baseline and scenario GHG emissions, and to identify trade-offs and uncertainties.

• A *Fire Tolerant* scenario included post-fire restoration with planting of climatically suitable fire-resistant species and salvage harvest in place of clearcut harvest.

## Graphical abstract

**Figure:**
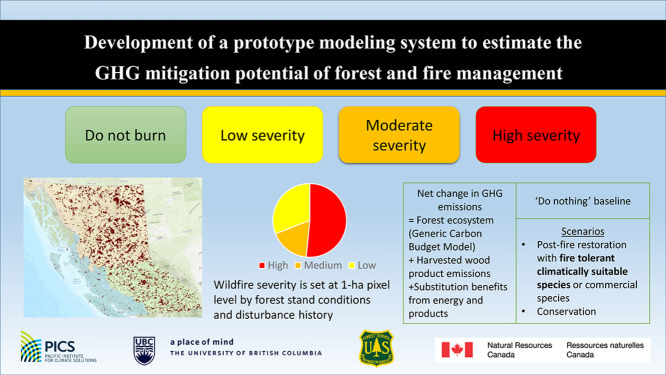

Specifications table

**Table:** 

Subject area:	Environmental Science
More specific subject area:	Forest sector Carbon modeling on climate change mitigation
Name of your method:	Prototype modeling system to estimate the GHG mitigation potential of forest and wildfire management
Name and reference of original method:	Modification of the Generic Carbon Budget Model (GCBM), which uses the same structure, equations, logic, and default assumptions of the Carbon Budget Model of the Canadian Forest Sector (CBM-CFS3 [20]) Kurz, W.A., Dymond, C.C., White, T.M., Stinson, G., Shaw, C.H., Rampley, G.J., Smyth, C., Simpson, B.N., Neilson, E.T., Trofymow, J.A., Metsaranta, J., Apps, M.J., 2009. CBM-CFS3: A model of carbon-dynamics in forestry and land-use change implementing IPCC standards. Ecol. Model. 220, 480-504.
Resource availability:	N.A.

## Method details

Efforts to reduce the rate of increase in atmospheric carbon dioxide (CO_2_) concentration require both a reduction in emissions and additional removals of CO_2_ from the atmosphere. However, increases in emissions from large wildfires in recent years have both directly emitted CO_2_ and other greenhouse gases to the atmosphere, and reduced sinks from forests in Canada [11]. Severe wildfire seasons burned roughly 1 Mha yr^−1^ in the western province of British Columbia (BC) in 2017, 2018, 2021 [2,3], and more severe wildfire seasons are expected in the future for the interior and southern Cordillera due to high fuel loads from Mountain Pine Beetle-caused tree mortality [40], and decades of fire exclusion and fire suppression resulting in widespread changes in forest stand composition, structure, fuel loads and fire regimes relative to historical conditions [5,8]. As well, climate change exacerbates the risk of wildfire, with increasing temperatures [13,41], and reduced fuel moisture [42]. In 2017, extreme warm and dry conditions increased the burned area by a factor of 7 to 11 [18], and an increase in burned area is predicted [6].

Given the anticipated increase in wildfire emissions, there is interest in understanding how forest management can identify optimal solutions to the trade-off between increasing carbon uptake and storage in forests, which can increase fuel loads for fire, and wildfire risk mitigation through post-fire salvage and planting of fire tolerant species, or other strategies such as reducing forest area or stand density to reduce fuel loads. The overall change in GHG emissions cannot be evaluated by examining the forest ecosystem alone. Optimal solutions for forest management must consider emissions and removals in the forest ecosystem, emissions from harvested wood products, including waste and post-consumer emissions, and potential substitution benefits of bioenergy in place of fossil fuel burning or use of wood products in place of emissions intensive materials [21,23,30]. It is of interest to compare multiple GHG reduction scenarios that include impacts of future wildfires and sustainable forest management at a provincial scale to support policy development.

We developed a prototype integrated C modeling system to simulate restoration of forests after wildfires and planting of fire tolerant species, and compared this strategy to a ‘do nothing’ after wildfire baseline, and other management strategies (restoration for timber, conservation). Our work builds on previous analyses of GHG reduction strategies [36] and future wildfire risk and restoration [27], with an incremental improvement that wildfire severity is modified by stand characteristics and disturbance history. Sample simulations were run for 50 years (2020 to 2070), which consider the short-term carbon losses associated with implementing activities (salvage harvest and residue treatment or changing forest species) with long-term gains from reducing wildfire severity and GHG emissions.

We validated methods by providing sample results including changes in wildfire emissions and associated drivers, changes in species cover, and net change in GHG emissions. This prototype system allowed us to identify data and knowledge gaps that must be addressed to reduce uncertainty in the net GHG impact for activities affected by wildfire risk.

### Forest ecosystem modeling

The forest ecosystem C balance was simulated using the Generic Carbon Budget Model (GCBM), which uses the same structure, equations, logic, and default assumptions of the Carbon Budget Model of the Canadian Forest Sector (CBM-CFS3 [20]), but in a spatially explicit modelling environment. Model simulations were from 1990 to 2070 for 61.5 Mha of public forests at 1 ha resolution. Forest inventory data and yield curves were provided by BC Ministry of Forests. The 2015 forest inventory was rolled back to represent the age structure of forests in 1990 [29]. Forest management activities occur only in areas eligible for harvest in the Timber Harvesting Land Base (THLB), which is 36% of forest area. The historical period used observed harvest, wildfire, and insect disturbance data. Historical wildfires were modeled as high severity stand-replacing fires. Harvest projections started in 2020, but no insect outbreaks were projected. Please see Smyth et al. [36] for additional details.

### Projected area burned

Wildfire projections from 2020 to 2070 used annual area burned layers based on Metsaranta et al. [27]. Their statistical approach to wildfire projections fit log-normal distributions to the observed area burned from 1950 to 2018 to generate future area burned forecasts for 100 Monte Carlo simulations. They further assumed that burned area would double over time (by 2070), and linearly increased the mean while holding the standard deviation constant. The maximum annual area burned was limited to twice the historical maximum and was assumed to double over time. Individual fires were generated from a second log-normal distribution that was fit to the fire size data and assumed a minimum of 100 ha. Fires were randomly distributed assuming an ellipsoidal shape and no re-burning for 10 years. Five 50-year annual burned area layer stacks were selected for the present study, corresponding to simulations for the 10^th^, 25^th^, 50^th^, 75^th^ and 90^th^ percentiles of the cumulative area burned between 2020 and 2070 (7.7, 8.2, 10.4, 12.3 and 15.1 Mha, respectively).

**Fig. 1:**
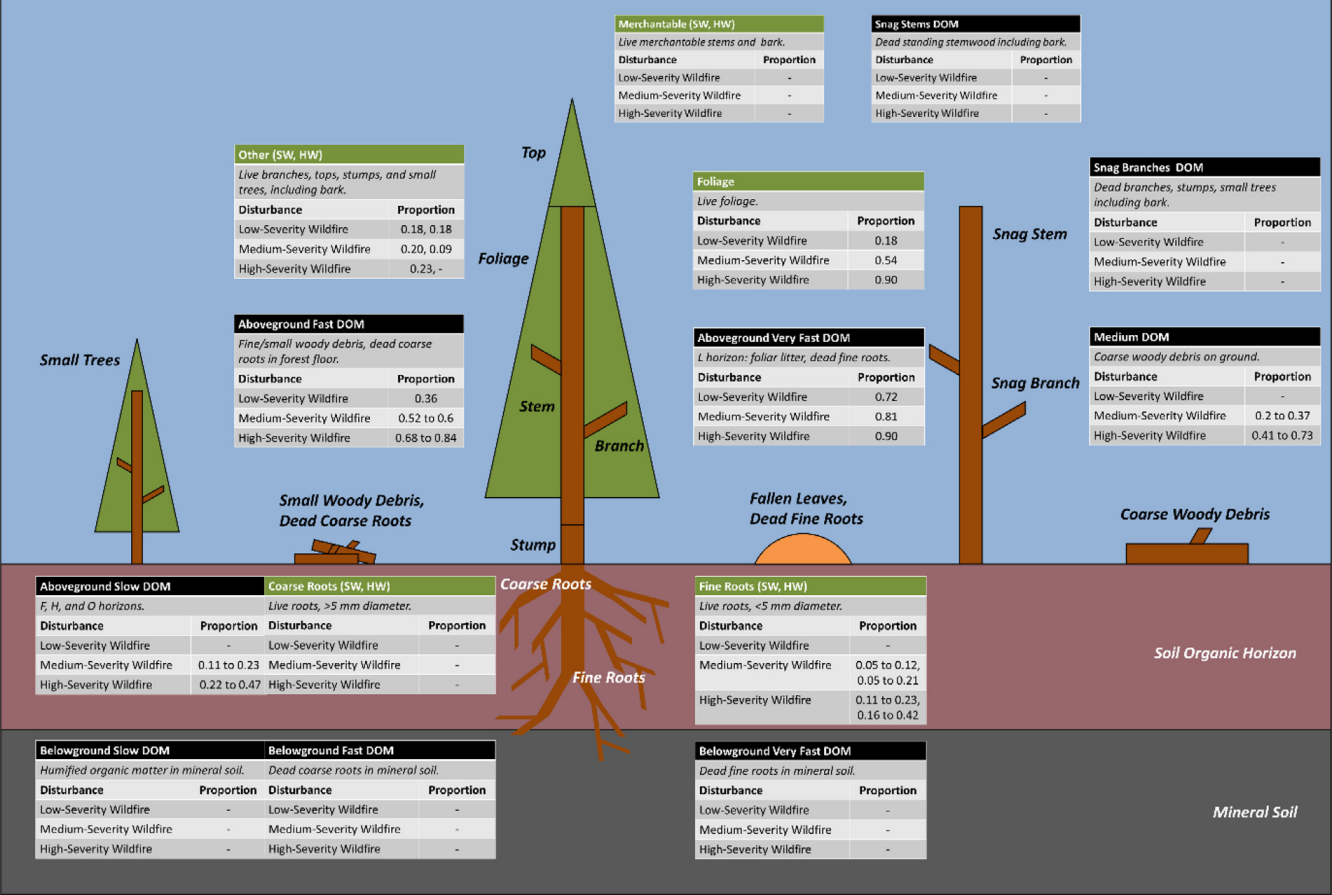
Wildfire transfer coefficients are shown for selected live (green) and dead (black) Generic Carbon Budget Model pools. Each table corresponds to a source pool and records the proportion of the C in that pool that is released to the atmosphere as CO_2_ for three wildfire severities: high, medium, and low. The range in values reflects the five ecozones within British Columbia.

A prototype system was developed to assign future wildfire severity to one of three classes (low, medium, and high) based on forest stand characteristics and disturbance history, as described in the Subsection on Wildfire Severity Controller. Wildfire emissions were modeled in GCBM using a disturbance matrix, which defines how C is transferred from one pool to other pools due to a disturbance [20], for each fire severity class and ecozone. Fig. 1 displays wildfire emission proportions from source C pools to the atmosphere as CO_2_ (please see the supplementary file for additional matrix coefficients, including non-CO2 emissions). High severity wildfire matrix coefficients were adapted from National Inventory Reporting [11], and medium and low disturbance matrix values were developed based on stand mortality percentages for inventory updates for BC: 50% mortality for medium, and 20% for low severity wildfires (https://catalogue.data.gov.bc.ca/dataset/fire-burn-severity-historical). To estimate direct fire emissions in units of CO_2_e, we applied 100-year Global Warming Potentials of 25 for CH_4_ and 298 for N_2_O [17].

### Future *Baseline* and scenarios

The forward-looking baseline and three management scenarios were developed starting in 2020 and through to 2070, Table 1. The *Baseline* scenario had no post-wildfire activities and included projected clearcut harvest with residues that were piled and burned (often referred to as slashpile burning) with more slashpile burning in the interior regions and less in the coastal region [36]. The three management scenarios included restoration of burned areas after wildfires (salvage harvesting, managing harvest residues and replanting seedlings), and reduced clearcut harvest levels relative to the *Baseline*. Post-fire restoration occurred after medium or high severity wildfire, if stands were accessible (within the timber harvesting landbase and within 500 meters of a road), suitable (medium to high site productivity, and conifer leading), and over 40 years old. Young stands (<40 years old) disturbed by high severity fire were under-planted instead of salvage harvested.

**Table 1:** Scenario descriptions and assumptions.

Scenario	Summary	Description
*Baseline* (Base)	No post-fire salvage or wildfire risk-abatement activities.	Post-harvest areas replanted with timber species. No post-fire salvage harvest.
*Fire Tolerant* (FrTol)	Post-fire restoration with fire-resistant species, and reduced clearcut harvest due to salvage harvest.	Restoration after medium or high severity wildfires for suitable stands (within the harvesting landbase, medium to high site productivity and conifer leading). Planted with climatically suitable species, with fire resistant and genetically modified seed preferred.
*Timber* (Tmbr)	Post-fire restoration with commercial species, and reduced clearcut harvest due to salvage harvest, and harvest residues for bioenergy.	Planted with commercial timber species. A portion of salvage harvest residues collected for bioenergy.
*Conservation* (Cons)	Reduced clearcut harvest of old stands. Post-fire restoration with commercial species.	Conserve a portion of old stands from harvesting, where ‘old’ is defined based on the natural disturbance type interval.

The wildfire-risk reduction scenario, *Fire Tolerant* (FrTol), included restoration of burned areas with salvage harvest, residue management of piling and burning, and planting climatically suitable seedlings with high fire resistance/resilience. The second scenario, *Timber* (Tmbr), included salvage harvest and planting of commercial timber species, and a portion of salvage harvest residues were collected for bioenergy. Harvest volumes were kept similar to *Baseline* levels by using salvage harvest to reduce clearcut harvest areas in the *Fire Tolerant* and *Timber* scenarios. The third scenario, *Conservation* (Cons), had post-fire restoration similar to the *Timber* scenario, except there was no collection of residues for bioenergy, and clearcut harvest levels were reduced by conserving old growth stands based on the *Restricted Harvest* scenario by Smyth et al. [36] and were not affected by salvage harvest levels.

The sudden shifts in harvest volumes due to large areas burned in some years were mitigated by reducing clearcut harvest to compensate for increased salvage harvest levels in the *Fire Tolerant* and *Timber* scenarios. A two-step process was used, where the scenarios were initially simulated with all of the clearcut and salvage areas, then area reductions to the clearcut spatial layers were implemented to compensate for salvage harvest, and then the simulations were rerun. Area reductions were estimated from average harvested merchantable C densities for clearcut and salvage harvest, estimated separately for coastal, northern and southern interior regions. In some rare cases, harvest area reductions to compensate salvage logging could exceed annual regional clearcut harvest area, in which case reductions in harvest rates were applied to subsequent years.

Post-fire planting for the *Timber* and *Conservation* scenarios used managed yield tables for lodgepole pine, Douglas-fir, spruce, and western larch (based on [27]), whereas planting in the *Fire Tolerant* scenario selected climatically suitable species, and fire resistant/genetically modified seed from the available selection of yield tables. High fire-resistant/resilience species (Table 1) were based on fire management stocking standard guidance, with preferred planting of seeds that were improved through seed selection programs (Douglas-fir, western larch, ponderosa pine) instead of regular seed (whitebark pine, balsam poplar [and aspen], and black cottonwood) [12]. Climatically suitable species were selected from available candidate yield tables (based on biogeoclimatic (BEC) zone, site productivity and leading species) and considered climate change-induced species selection indicators for zonal (01 or 101) sites for three future 20-year time periods (2021-2040, 2041-2060, 2051-2070) [26] (Table S1).

### Wildfire severity controller

Wildfire severity for future fires was assigned based on disturbance history and stand characteristics. A prototype disturbance controller module applied conditional rules on a per-pixel basis according to forest stand attributes (stand age, species and fuel loading) at the time of the fire, and the disturbance history. Please see Supplemental file 2 for a full description of the disturbance controller code. The applied rules approximate impacts of wildfire in response to stand conditions, where disturbance history and fuel loading can influence wildfire-severity, or even avoid burning. Table 2 describes the six conditional rules and the order in which the rules were applied. Once a rule is applied, the fire severity is set and subsequent rules are ignored, and if none of the conditional rules apply, the severity of the wildfire was assigned to medium.

**Table 2:** Rules applied to wildfire events to assign the wildfire severity depending on forest characteristics (age, leading species and coarse woody debris (CWD)), and disturbance history. Rules were applied in order, as indicated by the numbers. Wildfire severity was assigned to medium if none of the conditions were met.

Prevent burning	Low Severity	High severity
1. High severity burn within 10 years	3. Low severity burn within 20 years	2. Softwood stand undisturbed by fire for >= 80 years
	4. Medium severity burn within 20 years	6. High CWD
	5. Planted fire tolerant species or hardwoods (30 to 60 years old)	
	6. Low CWD	

The first rule prevented stands from burning if a stand-replacing fire had burned within 10 years, assuming that the previous high severity fires reduced fuel loads and that most fire-killed trees are still standing, and that little regrowth has occurred. The second rule set fire severity to high for softwood stands older than 80 years. The third and fourth rules set the fire to low severity if the stand had been disturbed by low or medium severity fires within 20 years. The fifth rule applied low severity fires to stands that had selected hardwood species (aspen, poplar, and cottonwood) between 30 and 60 years old, or to selected planted fire-tolerant softwood stands (ponderosa pine, whitebark pine, western larch, and Douglas-fir). The fire-tolerant species were selected from BC stocking guidance as having high fire resistance/resilience [12], and also included aspen. The sixth and final rule changed fire severity based on the level of coarse woody debris (CWD), which we use to approximate surface fuel loading. Wildfires were set to high severity if the CWD level was >20 tC ha^−1^ and were set to low severity fire if CWD level was <12 tC/ha. Fire severity rules based on levels of fine woody debris could not be constructed because the model aggregates several detrital pools together (dead coarse roots and small trees, fallen branches, dead tops and stumps of merchantable-sized trees).

### Results indicators

Results indicators for validation include direct wildfire emissions and area burned by severity class, changes in these indicators for each scenario relative to the *Baseline* as well as impacts on species cover, and the net change in GHG emissions (annual and cumulative) compared to the *Baseline*. The net GHG emissions included changes in the forest ecosystem emissions and removals, changes in emissions from harvested wood products, and their associated substitution benefits from using harvest residues for bioenergy and avoiding fossil fuel burning, and the use of wood products in place of emissions-intensive materials. We used the same model for tracking C in products as Smyth et al. [36] and the same set of low substitution benefit factors for products. The bioenergy substitution benefit factor was assumed to be 0.5 tCO_2_e of fossil emissions avoided per 1 tCO_2_e of bioenergy produced from burning harvest residues in bioenergy facilities [34].

## Method validation

The impact of the prototype fire severity rules was assessed by estimating the share of the cumulative area burned from 2020 to 2070 for each severity class for five sample Monte Carlo draws of wildfire futures. For the *Baseline* scenario, the shares of the cumulative area burned were 63% (minimum 58%, maximum 65%) for high severity fires, 15 (14, 16)% for medium, and 22 (20, 27)% for low. Average direct emissions from BC wildfires were 44 tC ha^−1^, which is higher than average emissions for Canada (up to 35 tC ha^−1^) from an atmospheric inversion system [46] which includes non-tree cover areas in their estimate.

The scenarios only affect the landbase eligible for timber harvest (THLB), which includes approximately 1/3 of the forest land base, and from this point onwards, we report validation results only for these areas. The wildfire severity shares of the cumulative area burned within the THLB was found to be shifted towards lower severities: high 47% (minimum 42%, maximum 51%), medium 23 (21, 25)%, and low 29 (25, 37)% for the *Baseline*.

The impact of the individual fire severity rules was quantified to understand which rules had the greatest impact. High severity fires were selected because of forest composition (older softwood stands over the age of 80, rule 2) which affected 26% (minimum 24%, maximum 28%) of the cumulative area burned, and because of high levels of coarse woody debris (CWD) 22 (18, 25)% (rule 6). Low severity wildfires were selected because of low levels of CWD for 22 (20, 26)% of the cumulative burned area, and because of planted fire-tolerant stands (rule 5) which affected 6 (5, 8)%.

Direct emissions were 40 tCO_2_e ha^−1^ (37 tCO2e ha^−1^ and 47 tCO_2_e ha^−1^ for 10^th^ and 90^th^ percentiles) for low severity wildfires, 120 (108, 121) tCO_2_e ha^−1^ for medium severity wildfires, and 243 (238, 256) tCO_2_e ha^−1^ for high severity wildfires.

**Fig. 2:**
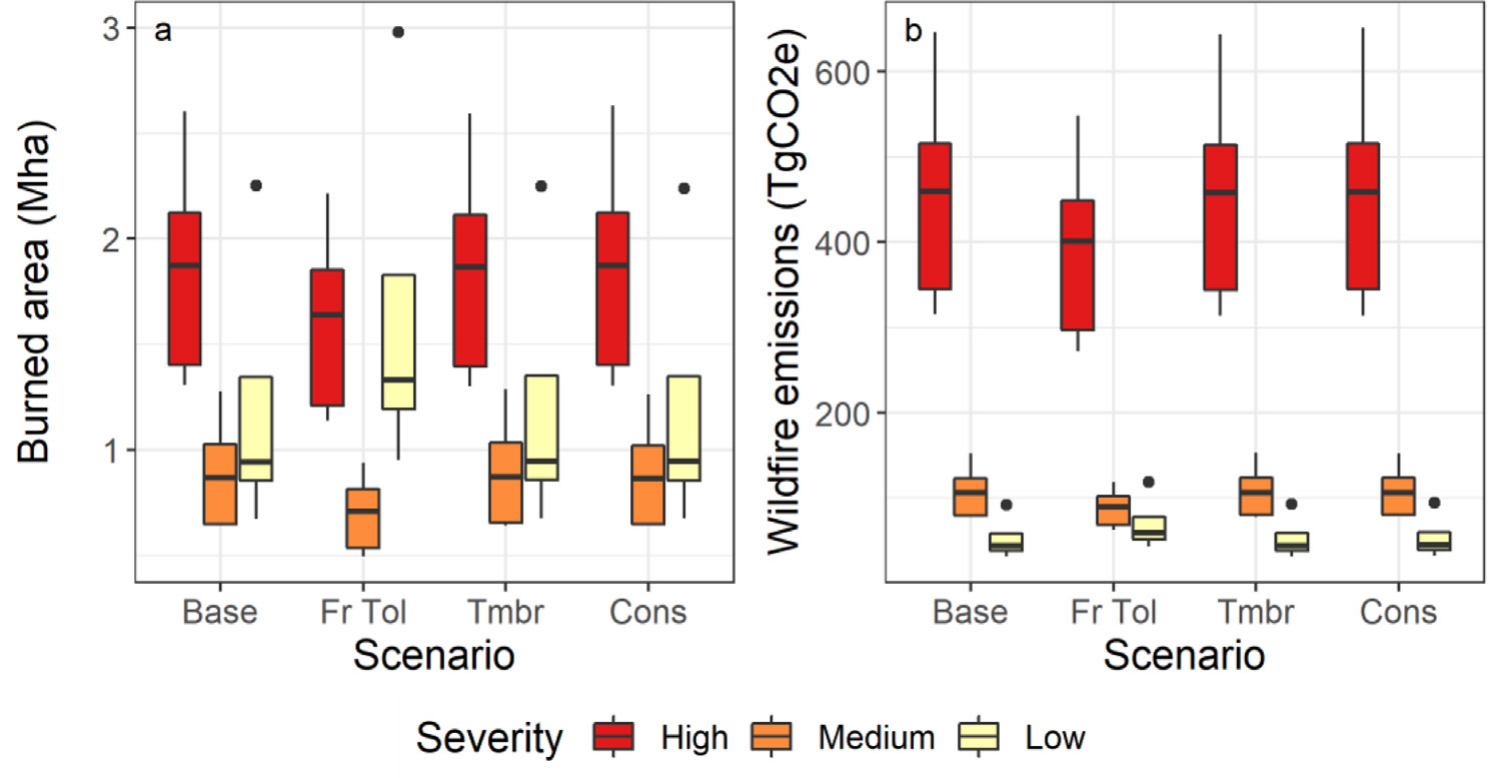
Cumulative (2020 to 2070) (a) area burned and (b) direct wildfires emissions by wildfire severity class for stands within the forest areas eligible for harvest. Sample results for model validation are shown for five draws, with boxplots identifying median (horizontal line), 25^th^ and 75^th^ percentiles (hinges). The whiskers extend 1.5 times the inter-quartile range from the hinges, and data points outside this range are plotted individually. Scenarios include the *Baseline* (Base), *Fire Tolerant* (FrTol), *Timber* (Tmbr) and *Conservation* (Cons).

**Fig. 3:**
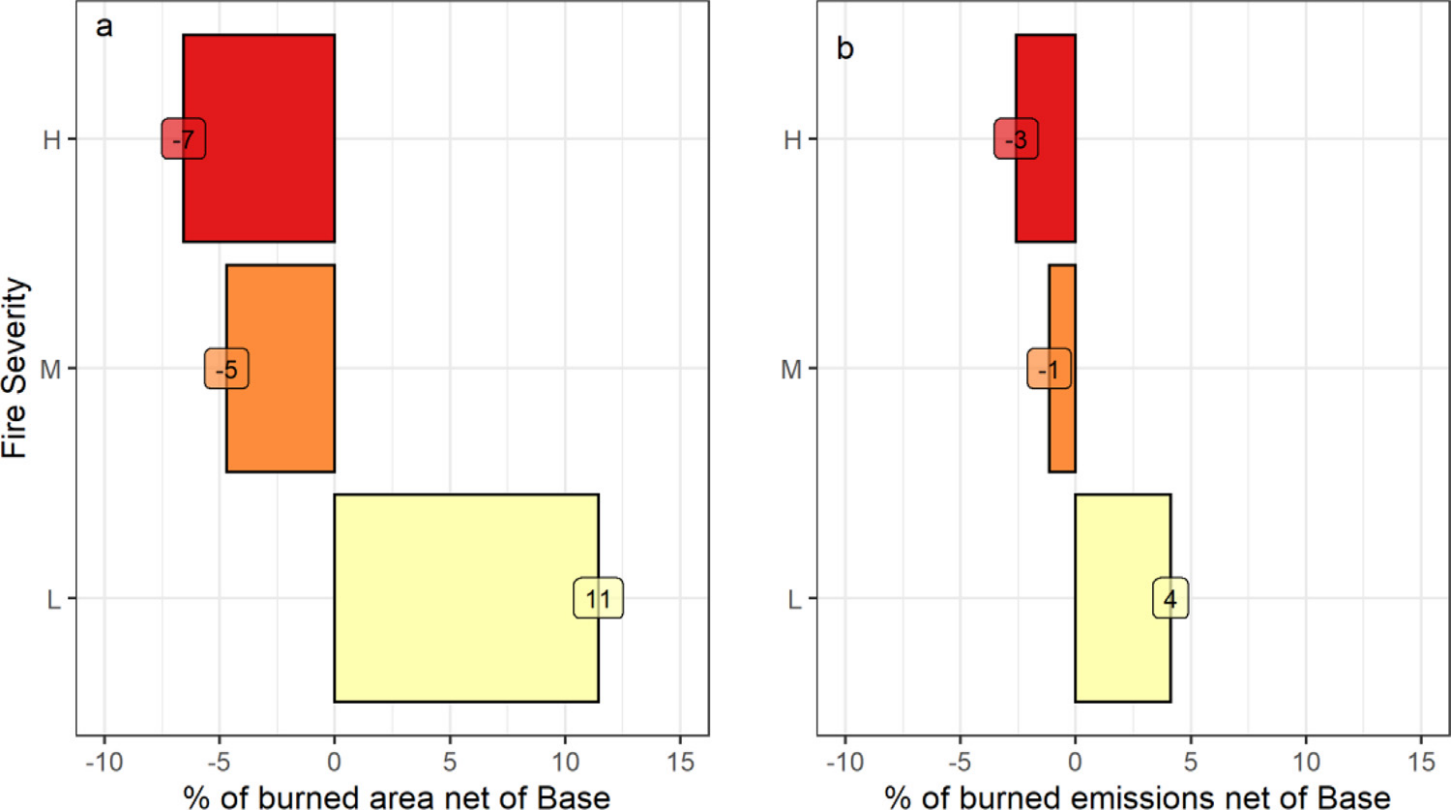
Differences in the percent of cumulative burned area by severity class (High, Medium, Low) in 2070 for the *Fire Tolerant* scenario, relative to the *Baseline*. There was 6% less area burned by high severity wildfires (min. -6%, max. -6%), and more area was burned with a lower severity wildfire 11% (min. 11%, max. 12%). Sample results are shown for model validation only.

**Fig. 4:**
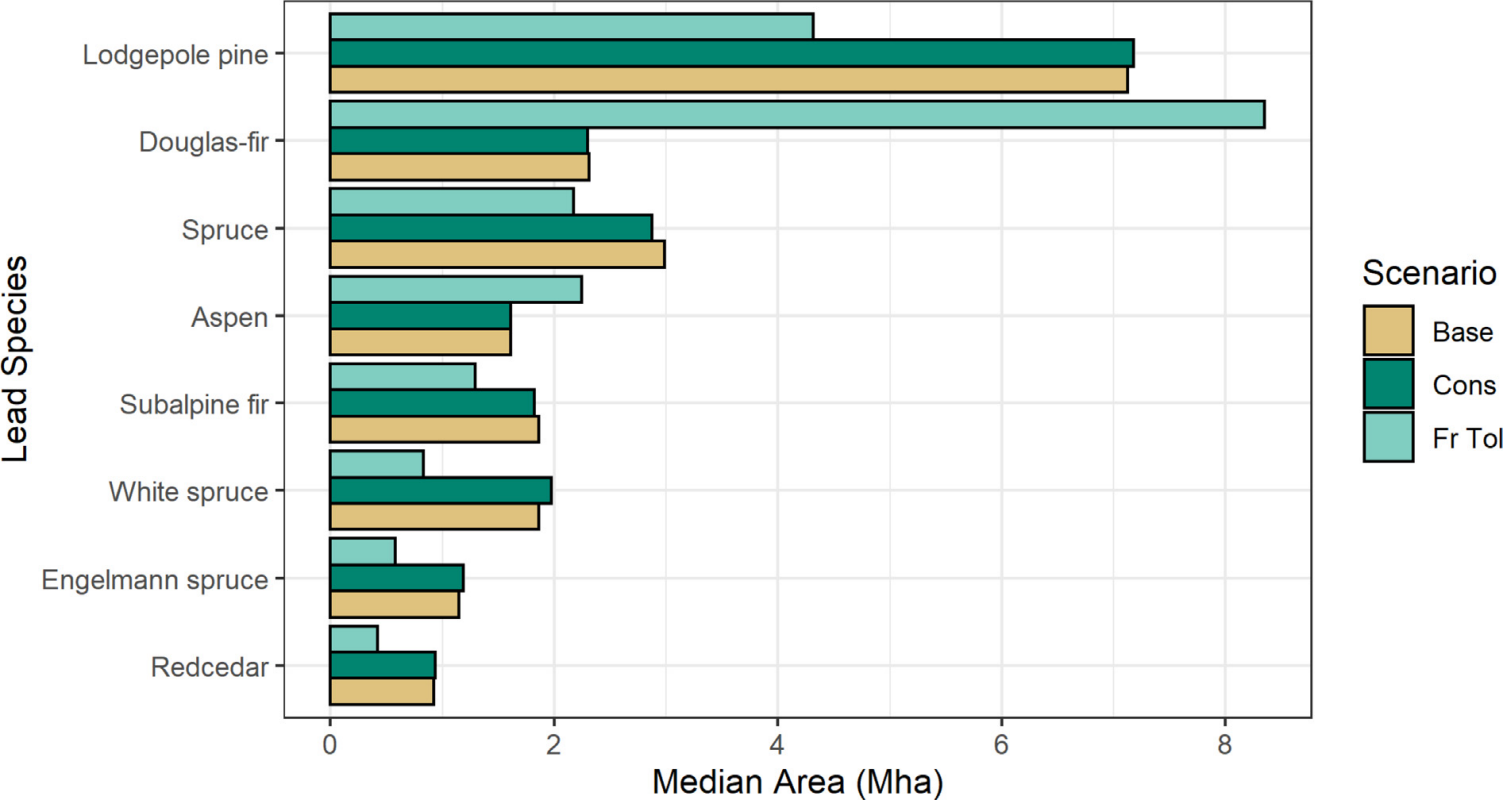
A subset of stand leading-species distribution for the *Baseline, Fire Tolerant* and *Conservation* scenarios in 2070. The *Fire Tolerant* scenario planted climatically suitable stands selected from high fire resistant species of Douglas-fir, western larch, ponderosa pine (all genetically improved seed), whitebark pine, balsam poplar, aspen, and black cottonwood. Sample results are shown for model validation only.

The *Fire tolerant* scenario, which planted high fire resistant species, had less area burned by high and medium severity wildfires (-6.8% [min -7.1%, max -6.7%] and -4.9 [-5.7, -4.3]% respectively), and more area 12 (11, 13)% burned with low severity wildfires relative to the *Baseline*. These reductions in wildfire severity resulted in lower direct emissions from wildfires. The other two scenarios had negligible impacts on the area burned by severity class. Planting of high fire-resistant species changed the species distribution for the *Fire Tolerant* scenario and resulted in more Douglas-fir and aspen stands and fewer lodgepole pine, spruce and western red cedar stands. Fig. 2 shows the cumulative (2020 to 2070) area burned and direct wildfire emissions for all scenarios by fire severity class, and Fig. 3 shows the change (from the *Baseline*) in the cumulative area burned and emissions by severity class for the *Fire Tolerant* scenario. Fig. 4 shows the species distribution in 2070 for this scenario compared to the *Baseline* and *Conservation* scenarios for a subset of the top species, and Table S2 provides the complete species list. Reduced levels of clearcut harvest in the *Conservation* scenario resulted in retention of species in older stands and less planting of post-harvest commercial species lodgepole pine, Douglas-fir, spruce, and western larch.

**Fig. 5:**
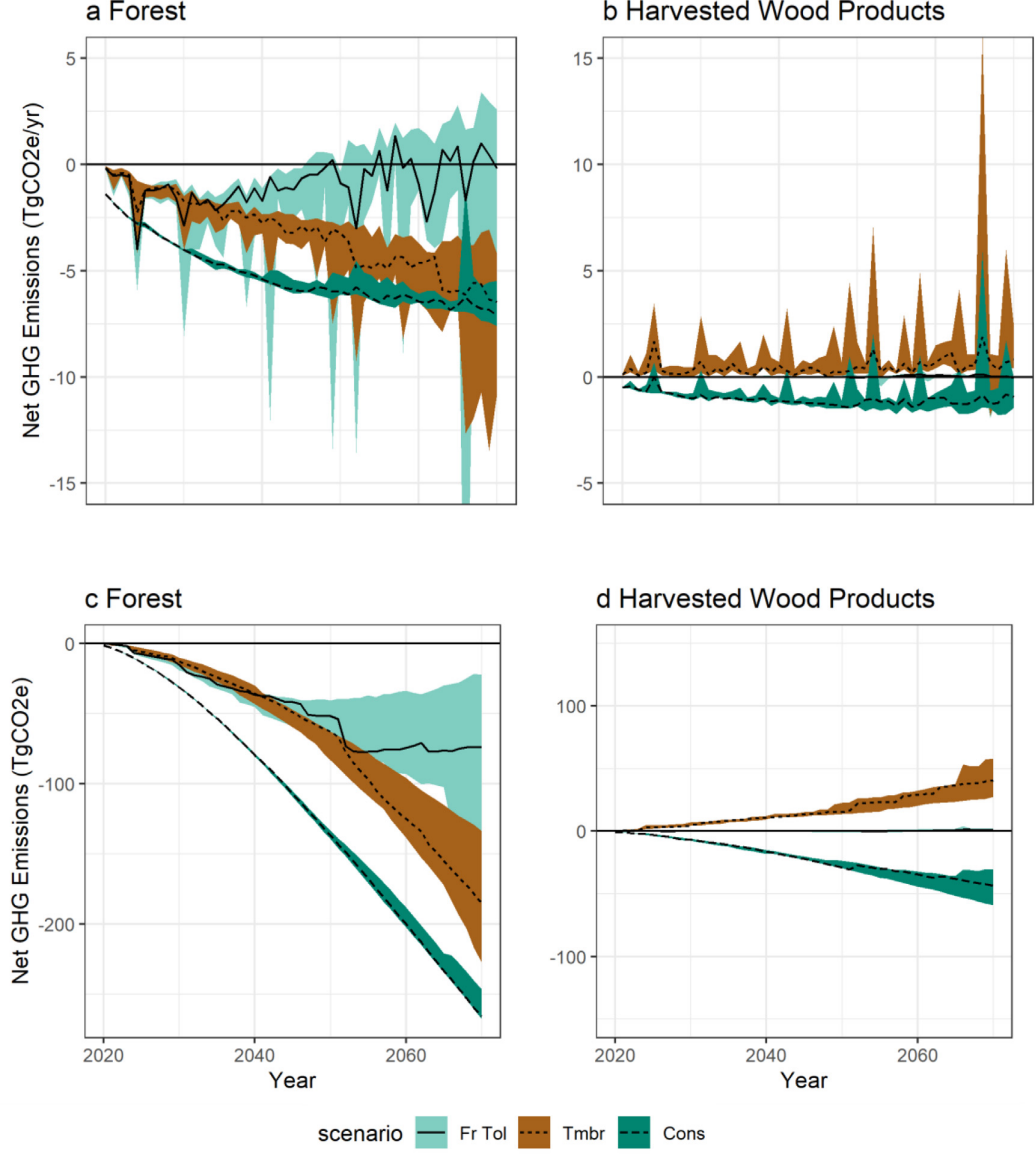
Net change in GHG emissions relative to the *Baseline* annual (a) forest and (b) harvested wood product component, and (c-d) cumulative timeseries for the median (lines) and 90^th^ percentiles (ribbon). A negative sign indicates a smaller source of emissions or greater removals, relative to the *Baseline* scenario. There were no net changes in GHG emissions before 2020 because activity implementation did not start until 2020. Sample results are shown for model validation only.

Fig. 5 shows the annual and cumulative net change in GHG emissions for the forest ecosystem and harvested wood product emissions that were estimated by subtracting *Baseline* emissions from each of the scenarios. Individual results for all five draws are shown in Figs. S2, S3, and S4.

The *Fire Tolerant* scenario had fewer emissions in the forest ecosystem in the short-term relative to the *Baseline*, but trended towards an increase in emissions in later decades. There was high variability between draws and the trends were punctuated by large net decreases in emissions during years with large burned areas. Net changes relative to the *Baseline* reflect three drivers: reduced mortality rates and fewer direct wildfire emissions because of fire-tolerant species rules (Fig. 2), avoided clearcut harvest but increased salvage harvest, and changes in post-salvage regrowth. Net emissions in the harvested wood product component for the *Fire Tolerant* scenario were negligible due to the objective of constant harvest levels, where reduced clearcut harvest balanced salvage harvest (See also Fig. S1).

The *Timber* scenario had fewer emissions in the forest ecosystem component, with a general trend of increasing annual sink, punctuated by moderate sinks during years with large burned area. Net changes reflect three drivers: avoided clearcut harvest but increased salvage harvest, a reduction in decay or slashpile burning emissions due to collection of harvest residues for bioenergy, and changes in post-salvage regrowth. Emissions from harvested wood products increased in the *Timber* scenario, relative to the *Baseline,* because collected harvest residues were burned for bioenergy and were assumed instantly oxidized.

The *Conservation* scenario had fewer emissions in the forest ecosystem and harvested wood product component than the *Baseline* because of reduced harvest levels. Both of the annual net GHG timeseries were punctuated by an increase in emissions due to salvage harvest after wildfires.

**Fig. 6:**
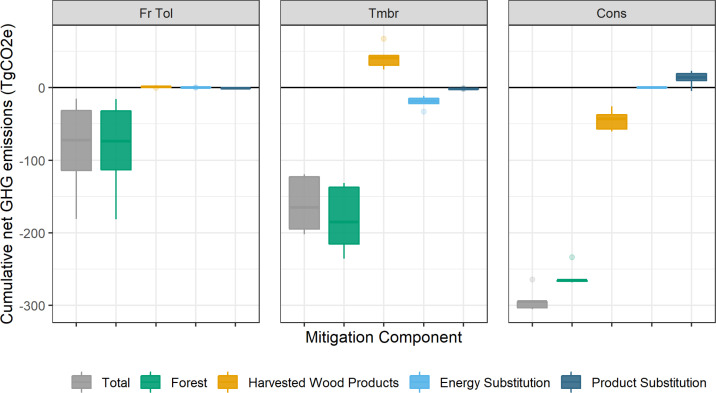
Cumulative net change in GHG emissions in 2070, relative to the *Baseline*. Sample results are shown for model validation only.

The two other components of net GHG changes, substitution benefits from bioenergy and wood products, are highly correlated, but opposite in sign, to net changes in the harvested wood product component. Fig. 6 shows the cumulative net GHG changes for all four components, as well as the total change in emissions. For the *Fire Tolerant* scenario, the only contribution to the net change in GHG emissions is from the forest component because harvest levels were unaffected. The other two scenarios had small contributions from non-forest components. The *Timber* scenario bioenergy emissions were partially offset by avoided fossil fuel burning, and the *Conservation* scenario reductions in harvest resulted in increased use of emissions-intensive materials.

## Rule Evaluation

Evaluation of the rule parameters is difficult due to the lack of information on fire severity, but the 2017 and 2018 fire severity and forest characteristics, and disturbance histories are available for comparison. Overall, burned areas observed in 2017 and 2018, had annual shares ranging from 10% to 44% for high severity fires, 40% to 57% for medium and 17% to 33% for low. If we compare these to the rule-based percentages simulated within the harvestable landbase (high 47% [minimum 42%, maximum 51%], medium 23 [21, 25]%, and low 29 [25, 37]%), we find that medium severity fires are under-represented in the model.

The first rule prevents a forest pixel from burning if a stand-replacing fire has occurred within 10 years. This rule was consistent with observations from the 2017 and 2018 fire seasons, where less than 1% of mapped fire areas burned in the decade before the fire (2007 to 2016). The second rule set fire severity to high for softwood stands older than 80 years based on earlier publications (e.g. [39]), and was consistent with observations that most of the high severity burned areas in 2017/2018 were in older stands; only 7% of high severity burned areas burned in stands younger than 60 years old. The third and fourth rules set the fire to low severity if the stand had been disturbed by low or medium severity fires within 20 years. This was based on Stevens-Rumann et al. [37] observing that burned landscapes mitigated fire impacts and subsequent fires had lower severity for several decades, and is a simpler representation of state transition model pathways [33]. Historical information on low and medium severity fires is not currently available, making it difficult to validate these rules.

The fifth rule set fire severity to low for selected hardwood species (aspen, poplar, and cottonwood) between 30 and 60 years old, and selected planted fire-tolerant softwood stands (ponderosa pine, whitebark pine, western larch, and Douglas-fir). For the 2017 fire perimeters, 8431 ha of Douglas-fir stands were affected by the wildfires, and 64% was either classified as unburned or low severity fire. For aspen stands between 30 and 60 years of age, 5570 ha were affected by the 2018 wildfires, and 45% was either classified unburned or low severity fire. Aspen's fire resistance ranking is listed as moderate and research and guidance indicates that generally broadleaf species are less flammable than other coniferous species and as a result may reduce fire behaviour [1,12]. Young seral aspen stands have been noted to avoid fires [4] because of low surface fuel loading, low crown volumes to sustain crown fires, and a lack of shade-tolerant understory trees to allow surface fires to move to the crown [9]. However, simulations have shown that high severity fires can still occur under severe and extreme fire weather [10].

The final rules changed fire severity based on the level of coarse woody debris (CWD), which we use to approximate surface fuel loading. Level thresholds were based on comparisons between fire severity in 2017 and 2018 and modeled 2016 CWD levels from Smyth et al. [34]. Stands under 60 years of age that burned with high severity fires had median CWD levels of 20 tC ha^−1^ and 24 tC ha^−1^, with 25^th^ and 75^th^ percentiles of 13 to 24 and 14 to 30 tC ha^−1^, for 2017 and 2018 fire seasons, respectively. Stands that burned with low severity had lower levels of CWD: 14 (11, 20) tC ha^−1^, and 12 (9, 23) tC ha^−1^ for 2017 and 2018 fire seasons, respectively.

Observed fire severity in 2017 and 2018 may have been affected by increased fuel loads due to mortality caused by Mountain Pine Beetle which killed lodgepole pine stands across 17 million hectares in the early 2000s. These stands with insect-killed trees were found to have faster fire spread and more crown fire than unaffected pine stands in a 2014 study [32]. For low levels of CWD, which we set to low severity fires, other studies have hypothesized that restoration treatments which reduce hazardous surface fuels (dead and down woody materials, litter, grasses, shrubs) through mechanical treatments and/or prescribed burning can shift fire events toward low severity surface fires [25].

### Limitations and uncertainties

In terms of uncertainties, we assumed that salvage harvest could reduce clear cut harvest and produce the same mix of wood products. Although we assumed lower salvage utilization rates for merchantable stemwood because of lower fibre quality, further investigation is needed on specific impacts affecting fibre quality such as the timing of the fire, fire temperature, or the time window for salvage operations [[47], [48]]. Previous anlysis on theoretical stands has shown that salvage logging may not fully compensate for timber losses to fire and eliminate fire-induced timber shortfalls, and depends on the stand age class distribution and burn rate [22]. Substitution benefits for wood products and bioenergy were assumed to be modest, and higher per-unit impacts have been assumed in earlier studies [24,35,45]. Harvested wood product emissions tracking with categorization of fibre quality and substitution based on end-use products is beyond the scope of the current study, but is considered in related research [43,44].

In terms of limitations, the currently implemented wildfire severity rules do not capture the dynamics of fire spread models [31,38], or the complexity of state transition models [33], but are meant to demonstrate the capability of a C model to adjust the fire severity based on pixel characteristics and disturbance history. There could be additional factors that influence fire severity such as topography and fire weather. Comparisons between satellite-derived differenced Normalized Burn Ratio (dNBR) and environmental controls on burn severity from historical wildfire will help identify important factors at the landscape scale. The extent to which reduced fuel loads (Fire Tolerant and Timber scenarios) reduce wildfire spread, or higher fuel loads (Conservation scenario) increase wildfire spread will affect the net carbon emissions and could alter the ranking of alternatives. This feedback between forest conditions and future wildfire behaviour is the subject of ongoing research.

In terms of the uncertainties, emissions associated with low severity and medium severity wildfires, simple ecozone-level burn and mortality proportion assumptions for modeled C pools have been developed based on existing stand-replacing wildfire impacts, but information from field studies or other modeling efforts is needed to improve GHG emissions estimates.

Sample results of net GHG emissions for five draws from 100 spatial time series of wildfire projections were presented, but these results are insufficient for a complete cost-benefit analysis, and many improvements are needed to reduce the uncertainty and better quantify the trade-offs and uncertainties for decision support. In the *Fire Tolerant* scenario, future growth of climatically suitable species was based on broad regional characteristics and a limited yield table library. Further, we did not consider post-fire regeneration failure in the *Baseline* scenario, or constraints in seedling availability for post-fire planting. Many refinements are possible to improve the ecological and climatic complexity [14,26], to ensure regeneration satisfies best practices, expectations and goals for reforestation set out by provincial land managers for sustainably managed forests on crown land. Desired data layers for improved modeling include spatially explicit layers of post-harvest or underplanting species mixes by decade, with associated growth expectations for different climate futures.

Many additional wildfire and forest management scenarios could be considered such as thinning, treating ladder fuels, prescribed burning, cultural burning, other fuel reduction techniques and altering landscape spatial configurations, as well as wildfire control activities [7,15,16,19,28]. Additional scenario analyses will also need to be informed by improving the modelled feedbacks between fuel characteristics and fire behaviour, both in terms of fire spread rates and fire intensity.

In conclusion, we have developed a prototype integrated C modeling framework that includes future wildfires that respond to forest stand characteristics and wildfire history and evaluated several simple management scenarios compared to a ‘do-nothing’ baseline. Comparing multiple scenarios within an integrated modeling framework will allow us to identify optimal solutions to complex trade-offs between short-term GHG losses associated with implementing mitigation measures and long-term gains in reduced wildfire emissions and improved resilience, or short-term gains from minimizing disturbances and long-term losses due to disturbance reversals, while considering society's demands for energy and products and their associated emissions. Improvements are needed to better represent fire severity rule sets and associated GHG emissions.

## Ethics statements

N/A

## CRediT authorship contribution statement

**C. Smyth:** Conceptualization, Methodology, Software, Formal analysis, Writing – original draft. **S.H. Xie:** Conceptualization, Methodology, Writing – review & editing. **T. Zaborniak:** Methodology, Software, Formal analysis, Writing – review & editing. **M. Fellows:** Methodology, Software. **C. Phillips:** Project administration, Writing – review & editing. **W.A. Kurz:** Conceptualization, Writing – review & editing, Funding acquisition.

## Declaration of Competing Interest

The authors declare that they have no known competing financial interests or personal relationships that could have appeared to influence the work reported in this paper.

## Data Availability

Code in attached file, data links in article.

## Appendix Supplementary materials

Image, application 1

Image, application 2

Image, application 3
