# Supplementary material for: Development of a prototype modeling system to estimate the GHG mitigation potential of forest and wildfire management
Source: MethodsX. 2022 Dec 23;10:101985. doi: 10.1016/j.mex.2022.101985 (PMC9841028; doi:10.1016/j.mex.2022.101985)
Supplement: Supplementary file 1 [file mmc1.docx]

**Disturbance Controller Description**

Supplemental File 2 to “Development of a prototype modeling system to estimate the GHG mitigation potential of forest and wildfire management”

Contents

[GCBM Disturbance Conditions 1](#_Toc113374519)

[Tiler-Attached Disturbance Conditions 1](#_Toc113374520)

[Example 1](#_Toc113374521)

[Rule-Based Disturbance Conditions 2](#_Toc113374522)

[Examples 4](#_Toc113374523)

[Complete JSON configuration 4](#_Toc113374524)

[Other JSON configuration examples 5](#_Toc113374525)

[Simulation configuration from modules_cbm.json 6](#_Toc113374526)

# GCBM Disturbance Conditions

The Generic Carbon Budget Model (GCBM) allows conditions to be applied to disturbance events which determine whether or the event should run, and if the disturbance type should be maintained or replaced.

Disturbance conditions are configured in two different ways: directly through the Tiler script with a more limited set of features, or indirectly through a JSON configuration file where conditions are matched to disturbance events by disturbance type and stand characteristics.

## Tiler-Attached Disturbance Conditions

These conditions are attached directly to disturbance events in the Tiler script in the same way as transition rules can be, and can only determine whether a disturbance event runs or not. A disturbance event can have one or more conditions which must all be satisfied in order for the disturbance to be applied.

In this mode, conditions are limited to comparing the value of any variable (i.e. age) or classifier to a target value using < (less than), == (equal to), or >= (greater than or equal to) operators based on the current state of a pixel at the time of disturbance.

Conditions are specified in the DisturbanceLayer constructor’s optional "conditions" parameter as a list of lists of items in the format:

["<variable name>", "<comparison operator>", <target>]

### Example

DisturbanceLayer(

rule_manager,

VectorLayer("disturbances", "disturbances.shp", [

Attribute("year"),

Attribute("dist_type")

]),

year=Attribute("year"),

disturbance_type=Attribute("dist_type"),

conditions=[

["age", "<", 30],

]

)

## Rule-Based Disturbance Conditions

Directly attaching conditions to disturbance events through the Tiler script is easy to understand and use for simple cases, but is sometimes cumbersome or too simple for more advanced projects. An alternative "soft matching" approach is also available, and works similarly to the way transition rules can be matched by classifier set and disturbance type.

Rule-based conditions still only apply to disturbances that exist within spatial layers – this feature does not generate disturbance events, only determines whether they should run or not, or be replaced by a different disturbance type depending on the specified criteria.

Configuration conditions have access to all pixel data: variables, including the classifier set, carbon pool values, the age of the stand at the time of the disturbance, and the complete prior disturbance history of the stand including the disturbance type.

A condition is composed of the name of one or more disturbance types and optionally one or more variables, classifiers, or pool values to match, and one or both types of event-modifying conditions: run conditions, which determine if the event runs at all, and override conditions, which replace the disturbance type with a different one.

If multiple conditions match to the same disturbance event using the outer (matching) criteria, the first one of each type that satisfies its inner conditions is applied – that is, if any run condition is met, the event runs; if any override condition is met, the disturbance type is replaced by the first satisfied condition. If an outer condition contains both run conditions and override conditions, the override conditions are skipped if the run conditions are not satisfied.

There are two possible event-modifying condition types, both of which can be added to a single rule:

"run_conditions" – a list of criteria that determine whether a disturbance event should run at all. Each top-level item in the list is a self-contained set of criteria; the disturbance event runs for the current pixel if and only if any of these sets of criteria is satisfied.

"override_conditions" – a list of criteria that determine when the disturbance type of an event should be replaced by another one (defined outside the conditions list as "override_disturbance_type"). Each top-level item in the list is a self-contained set of criteria, and if any one of these sets of criteria is satisfied, the disturbance event has its disturbance type replaced.

Disturbance event-modifying conditions are composed of one or more criteria, which can be either the name of a classifier, variable, or pool to compare to an exact value:

"LdSpp": "Pine"

Or a list of values:

"LdSpp": ["Pine", "Oak", "Spruce"]

Or a < or >= comparison:

"age": [">=", 10]

Or a "between" (inclusive values) comparison:

"age": ["<->", 20, 50]

Or a disturbance sequence, where the disturbance history to match is listed from most to least recent, with each item specified as a pair of [disturbance type, maximum years since next most recent event], where the years part is optional (empty or -1 = unlimited); the topmost item is relative to the current simulation year:

"disturbance_sequence": [

["Wildfire", 5],

["Wildfire", 20]

]

Sequence items can also be a 4-item list of [disturbance type, maximum years since most recent event, age comparison type, age at disturbance event]:

"disturbance_sequence": [

["Wildfire"],

["Wildfire", -1, ">=", 10]

]

Sequence items can also be a 5-item list of [disturbance type, maximum years since most recent event, "between" operator symbol, minimum age at disturbance event, maximum age at disturbance event]:

"disturbance_sequence": [

["Wildfire"],

["Wildfire", -1, "<->", 10, 25]

]

Or a collection of pools:

"SoftwoodMerch + HardwoodMerch": [">=", 25]

## Examples

### Complete JSON configuration

"CBMDisturbanceListener": {

"enabled": true,

"order": 3,

"library": "moja.modules.cbm",

"settings": {

"vars": [],

"conditions": [

{

"disturbance_type": "Natural Succession",

"run_conditions": [

{

"disturbance_sequence": [

["Wildfire_HighSev"],

["Wildfire_HighSev", 5]

]

},

{

"disturbance_sequence": [

["Prescribed burn - Surface"]

]

}

]

},

{

"disturbance_type": "Wildfire_HighSev",

"override_disturbance_type": "Wildfire_LowSev"

"override_conditions": [

{

"disturbance_sequence": [

["Wildfire_HighSev", 40]

],

},

{

"age": ["<", 30]

}

],

}

]

}

},

Condition 1:

This condition applies to Natural Succession disturbance events and has two run-time rules that read:

1. "Run this event if the most recent disturbance was a high-severity fire at any time before the current simulation year, preceded by another high-severity fire within at least 5 years."

*OR*

1. "Run this event if prescribed burn was the most recent disturbance at any time before the current simulation year."

Condition 2:

This condition applies to high-severity wildfire events and has two disturbance type override rules that downgrade the disturbance to low severity if:

1. There was a high-severity wildfire within 40 years of the current simulation year.

*OR*

1. The stand age is less than 30 years.

### Other JSON configuration examples

{

"disturbance_type": "Wildfire_MedSev",

"override_disturbance_type": "Wildfire_HighSev",

"override_conditions": [

{

"SoftwoodMerch + HardwoodMerch": [">=", 15]

}

]

}

This condition upgrades a medium-severity wildfire to high severity if the sum of the softwood and hardwood merchantable pools is at least 15 tC/ha.

{

"disturbance_type": "Ants",

"run_conditions": [

{

"Humidity": "Dry",

"Has_Anthills": "Yes"

}

]

}

This condition applies the Ants disturbance if any only if the Humidity classifier is "Dry" and the "Has_Anthills" classifier is "Yes".

{

"disturbance_type": [

"Mountain pine beetle — Severe impact",

"Mountain pine beetle — Very severe impact"

],

"age": [20, 50],

"run_conditions": [

{

"disturbance_sequence": [

["Mountain pine beetle — Low impact"],

["Mountain pine beetle — Low impact", 7]

]

}

]

}

This condition applies to severe and very severe mountain pine beetle events on stands between 20 and 50 years old. For these stands, the two most recent disturbances must have been a pair of low impact mountain pine beetle disturbances within 7 years of each other any number of years before the current simulation year. For stands outside that age range, the severe and very severe pine beetle disturbances run as normal.

{

"disturbance_type": "Wildfire – Moderate severity",

"run_conditions": [

{

"age": [">=", 20]

}

],

"override_disturbance_type": "Wildfire – High severity",

"override_conditions": [

{

"LeadingSpecies": "Black spruce"

}

]

}

This condition applies to all moderate severity wildfire events and restricts them to stands at least 20 years old, upgrading them to high severity if the leading species classifier is "Black spruce".

## Simulation configuration from modules_cbm.json

"conditions": [

{

"disturbance_type": [

"Clearcut harvest with slash pile burning - future",

"Clearcut harvest with salvage and slash pile burning at regional occurrence rates"

],

"override_disturbance_type": "Natural succession, type 1",

"override_conditions": [{"age": ["<", 40]}]

},

{

"disturbance_type": [

"Wildfire_MediumSev_future - accessible",

"Wildfire_MediumSev_future - inaccessible"

],

"override_disturbance_type": "Natural succession, type 2",

"override_conditions": [

{"disturbance_sequence": [["Thinning and prescribed burning", 10]]}

]

},

{

"disturbance_type": [

"Wildfire_MediumSev_future - accessible",

"Wildfire_MediumSev_future - inaccessible"

],

"override_disturbance_type": "Natural succession, type 3",

"override_conditions": [

{"disturbance_sequence": [["Wildfire_HighSev_future - accessible, type 1", 10]]},

{"disturbance_sequence": [["Wildfire_HighSev_future - accessible, type 2", 10]]},

{"disturbance_sequence": [["Wildfire_HighSev_future - inaccessible, type 1", 10]]},

{"disturbance_sequence": [["Wildfire_HighSev_future - inaccessible, type 2", 10]]},

{"disturbance_sequence": [["Wildfire_HighSev_historical", 10]]}

]

},

{

"disturbance_type": "Wildfire_MediumSev_future - accessible",

"override_disturbance_type": "Wildfire_HighSev_future - accessible, type 1",

"override_conditions": [

{

"LdSpp": [

"Douglas-fir", "Redcedar", "Spruce", "Western larch", "Engelmann spruce",

"White spruce", "Lodgepole pine", "Amabilis fir", "Tamarack", "Sitka spruce",

"Subalpine fir", "Western hemlock", "Western white pine", "Softwoods",

"Mountain hemlock", "Whitebark pine", "Ponderosa pine", "Black spruce",

"Balsam fir", "Cypress", "Alpine larch"

],

"disturbance_sequence": [["none", 80]]

}

]

},

{

"disturbance_type": "Wildfire_MediumSev_future - inaccessible",

"override_disturbance_type": "Wildfire_HighSev_future - inaccessible, type 1",

"override_conditions": [

{

"LdSpp": [

"Douglas-fir", "Redcedar", "Spruce", "Western larch", "Engelmann spruce",

"White spruce", "Lodgepole pine", "Amabilis fir", "Tamarack", "Sitka spruce",

"Subalpine fir", "Western hemlock", "Western white pine", "Softwoods",

"Mountain hemlock", "Whitebark pine", "Ponderosa pine", "Black spruce",

"Balsam fir", "Cypress", "Alpine larch"

],

"disturbance_sequence": [["none", 80]]

}

]

},

{

"disturbance_type": "Wildfire_MediumSev_future - accessible",

"override_disturbance_type": "Wildfire_LowSev_future - accessible, type 1",

"override_conditions": [

{"disturbance_sequence": [["Thinning and prescribed burning", -1]]}

]

},

{

"disturbance_type": "Wildfire_MediumSev_future - accessible",

"override_disturbance_type": "Wildfire_LowSev_future - accessible, type 2",

"override_conditions": [

{"disturbance_sequence": [["Wildfire_LowSev_future - accessible, type 1", 20]]},

{"disturbance_sequence": [["Wildfire_LowSev_future - accessible, type 2", 20]]},

{"disturbance_sequence": [["Wildfire_LowSev_future - accessible, type 3", 20]]},

{"disturbance_sequence": [["Wildfire_LowSev_future - accessible, type 4", 20]]},

{"disturbance_sequence": [["Wildfire_LowSev_future - accessible, type 5", 20]]}

]

},

{

"disturbance_type": "Wildfire_MediumSev_future - accessible",

"override_disturbance_type": "Wildfire_LowSev_future - accessible, type 3",

"override_conditions": [

{"disturbance_sequence": [["Wildfire_MediumSev_future - accessible", 20]]}

]

},

{

"disturbance_type": "Wildfire_MediumSev_future - accessible",

"override_disturbance_type": "Wildfire_LowSev_future - accessible, type 4",

"override_conditions": [

{

"age": ["<->", 30, 60],

"LdSpp": ["Aspen", "Poplar", "Cottonwood"]

},

{

"LdSpp": ["Ponderosa pine", "Whitebark pine", "Douglas-fir", "Western larch"],

"AU": ["<->", 3000, 6000]

}

]

},

{

"disturbance_type": "Wildfire_MediumSev_future - inaccessible",

"override_disturbance_type": "Wildfire_LowSev_future - inaccessible, type 1",

"override_conditions": [

{"disturbance_sequence": [["Thinning and prescribed burning", -1]]}

]

},

{

"disturbance_type": "Wildfire_MediumSev_future - inaccessible",

"override_disturbance_type": "Wildfire_LowSev_future - inaccessible, type 2",

"override_conditions": [

{"disturbance_sequence": [["Wildfire_LowSev_future - inaccessible, type 1", 20]]},

{"disturbance_sequence": [["Wildfire_LowSev_future - inaccessible, type 2", 20]]},

{"disturbance_sequence": [["Wildfire_LowSev_future - inaccessible, type 3", 20]]},

{"disturbance_sequence": [["Wildfire_LowSev_future - inaccessible, type 4", 20]]},

{"disturbance_sequence": [["Wildfire_LowSev_future - inaccessible, type 5", 20]]}

]

},

{

"disturbance_type": "Wildfire_MediumSev_future - accessible",

"override_disturbance_type": "Wildfire_LowSev_future - inaccessible, type 3",

"override_conditions": [

{"disturbance_sequence": [["Wildfire_MediumSev_future - inaccessible", 20]]}

]

},

{

"disturbance_type": "Wildfire_MediumSev_future - inaccessible",

"override_disturbance_type": "Wildfire_LowSev_future - inaccessible, type 4",

"override_conditions": [

{

"age": ["<->", 30, 60],

"LdSpp": ["Aspen", "Poplar", "Cottonwood"]

},

{

"LdSpp": ["Ponderosa pine", "Whitebark pine", "Douglas-fir", "Western larch"],

"AU": ["<->", 3000, 6000]

}

]

},

{

"disturbance_type": ["Wildfire_MediumSev_future - accessible"],

"override_disturbance_type": "Wildfire_HighSev_future - accessible, type 2",

"override_conditions": [{"MediumSoil": [">=", 20]}]

},

{

"disturbance_type": ["Wildfire_MediumSev_future - inaccessible"],

"override_disturbance_type": "Wildfire_HighSev_future - inaccessible, type 2",

"override_conditions": [{"MediumSoil": [">=", 20]}]

},

{

"disturbance_type": ["Wildfire_MediumSev_future - accessible"],

"override_disturbance_type": "Wildfire_LowSev_future - accessible, type 5",

"override_conditions": [{"MediumSoil": ["<", 12]}]

},

{

"disturbance_type": ["Wildfire_MediumSev_future - inaccessible"],

"override_disturbance_type": "Wildfire_LowSev_future - inaccessible, type 5",

"override_conditions": [{"MediumSoil": ["<", 12]}]

},

{

"disturbance_type": [

"Salvage logging after fire, residue capture",

"Salvage logging after fire, no residue capture"

],

"override_disturbance_type": "Natural succession, type 6",

"override_conditions": [

{"disturbance_sequence": [["Wildfire_LowSev_future - accessible, type 1", 1]]},

{"disturbance_sequence": [["Wildfire_LowSev_future - accessible, type 2", 1]]},

{"disturbance_sequence": [["Wildfire_LowSev_future - accessible, type 3", 1]]},

{"disturbance_sequence": [["Wildfire_LowSev_future - accessible, type 4", 1]]},

{"disturbance_sequence": [["Wildfire_LowSev_future - accessible, type 5", 1]]}

]

},

{

"disturbance_type": [

"Salvage logging after fire, residue capture",

"Salvage logging after fire, no residue capture"

],

"override_disturbance_type": "Natural succession, type 7",

"override_conditions": [

{"disturbance_sequence": [["Wildfire_MediumSev_future - accessible", 1, "<", 40]]}

]

},

{

"disturbance_type": [

"Salvage logging after fire, residue capture",

"Salvage logging after fire, no residue capture"

],

"override_disturbance_type": "Natural succession, type 5",

"override_conditions": [

{"disturbance_sequence": [["Natural succession, type 1", 1]]},

{"disturbance_sequence": [["Natural succession, type 2", 1]]},

{"disturbance_sequence": [["Natural succession, type 3", 1]]},

{"disturbance_sequence": [["Natural succession, type 4", 1]]},

{"disturbance_sequence": [["Natural succession, type 5", 1]]},

{"disturbance_sequence": [["Natural succession, type 6", 1]]},

{"disturbance_sequence": [["Natural succession, type 7", 1]]}

]

},

{

"disturbance_type": [

"Salvage logging after fire, residue capture",

"Salvage logging after fire, no residue capture"

],

"override_disturbance_type": "Underplanting",

"override_conditions": [

{

"disturbance_sequence": [

["Wildfire_HighSev_future - accessible, type 1", 1, "<", 40]

]

},

{

"disturbance_sequence": [

["Wildfire_HighSev_future - accessible, type 2", 1, "<", 40]

]

}

]

}

]
