# Supplementary material for: Development of a prototype modeling system to estimate the GHG mitigation potential of forest and wildfire management
Source: MethodsX. 2022 Dec 23;10:101985. doi: 10.1016/j.mex.2022.101985 (PMC9841028; doi:10.1016/j.mex.2022.101985)
Supplement: Supplementary file 2 [file mmc2.docx]

**Supplementary material *and/or* additional information [OPTIONAL]**

**Table S1.** Average suitability values by Biogeoclimatic (BEC) zone and selected species.

| BEC zone | BEC abbr. | Selected species | Suitability (ave.) |
| --- | --- | --- | --- |
|  |  |  | Primary |
| Bunchgrass | BG | Douglas-fir | 0.5 |
| Boreal White and Black Spruce | BWBS | Aspen | 0.7 |
| Coastal Douglas-fir | CDF | Douglas-fir | 1.0 |
| Coastal Western Hemlock | CWH | Douglas-fir | 0.7 |
| Interior Cedar-Hemlock | ICH | Douglas-fir | 0.8 |
| Interior Douglas-fir | IDF | Douglas-fir | 0.6 |
| Mountain Hemlock | MH | Douglas-fir | 0.7 |
| Montane Spruce | MS | Douglas-fir | 0.7 |
| Spruce – Willow – Birch | SWB | Aspen | 0.4 |
|  |  |  | Secondary |
|  |  |  |  |
| Engelmann Spruce – Subalpine Fir | ESSF | Douglas-fir | 0.5 |
| Ponderosa Pine | PP | Douglas-fir | 0.4 |
| Sub-Boreal Pine - Spruce | SBPS | Douglas-fir | 0.6 |
| Sub-Boreal Spruce | SBS | Western larch | 0.5 |

*
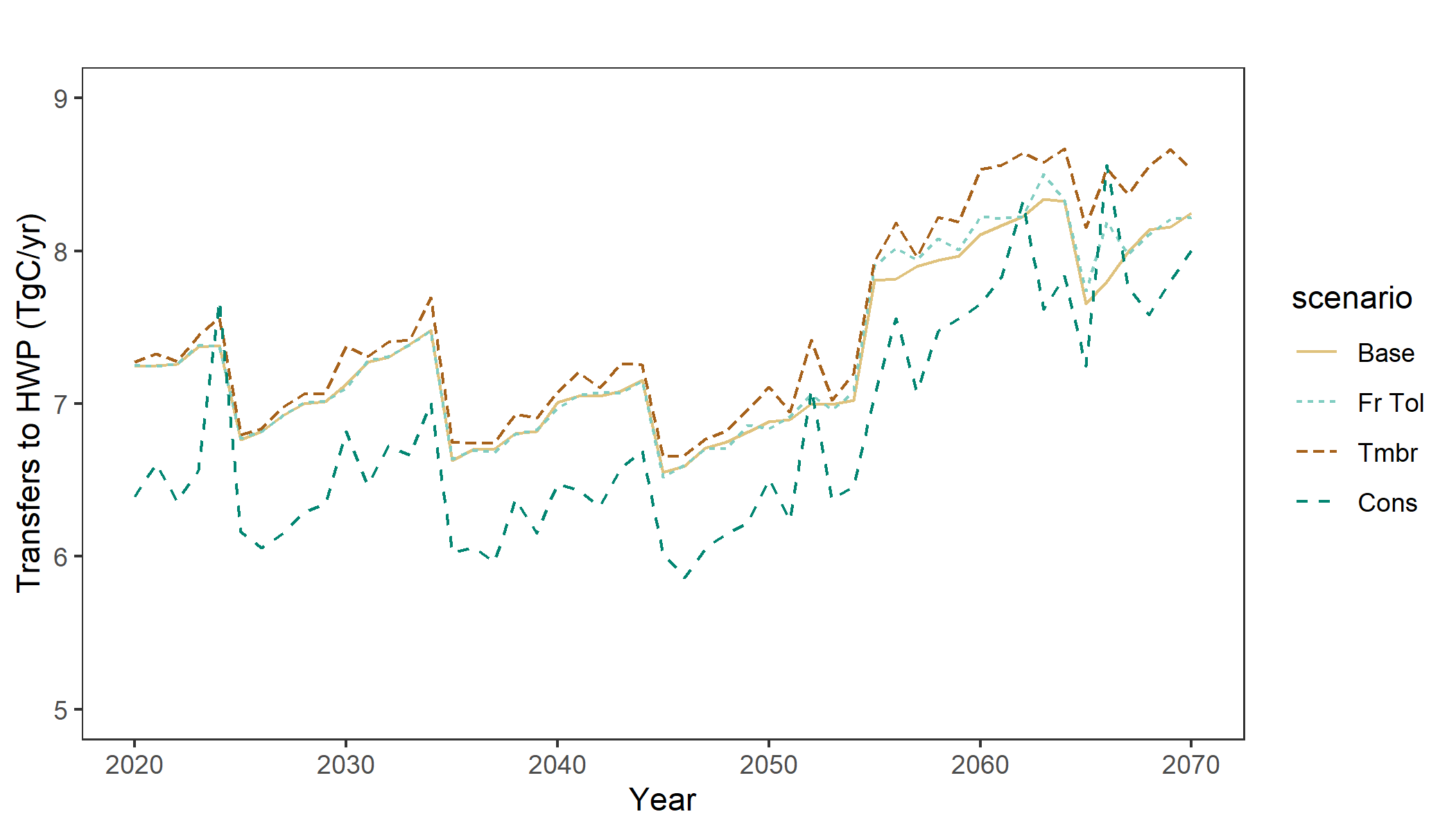
*

*
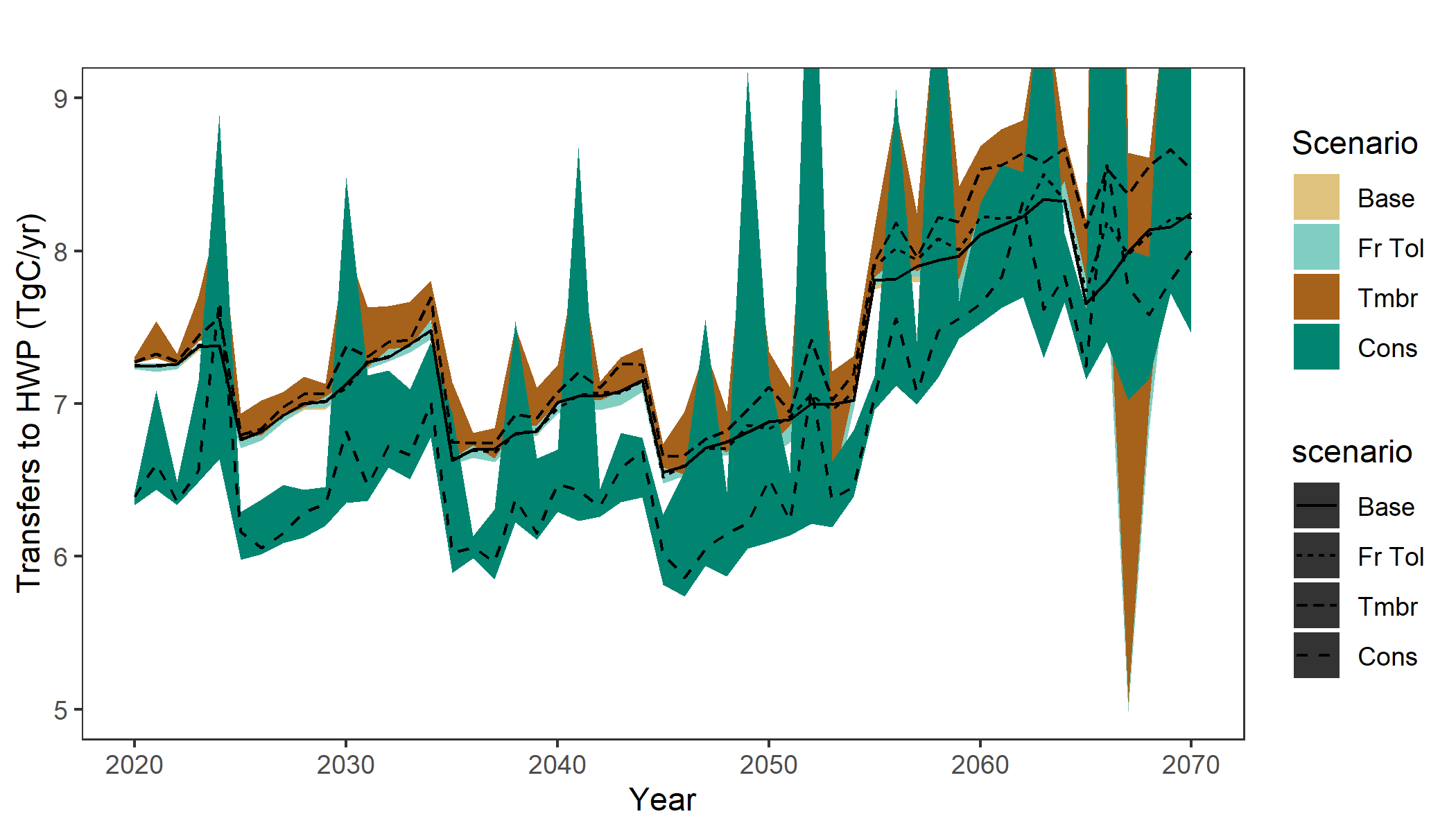
*

**Figure S1.** Annual time series of the C transferred to harvested wood products (top panel shows median timeseries, bottom panel shows median values with confidence interval ribbon). Overall, the *Conservation* scenario has the smallest amount of C transferred to wood products, even with post-fire salvage inputs. The *Baseline* with clearcut harvest had similar C transfers as the *Fire Tolerant* scenario, which included clearcut and post-fire salvage. The *Timber* scenario has higher C transfers to wood products because of the collection of harvest residues for bioenergy.

**Table S2.** Stand species distribution in 2070 for all scenarios and species (median over five draws).

| Leading Species | Area (Mha) | | | |
| --- | --- | --- | --- | --- |
|  | *Baseline* | *Conservation* | *Fire Tolerant* | *Timber* |
| Alpine larch | 0.004 | 0.004 | 0.003 | 0.004 |
| Amabilis fir | 0.189 | 0.181 | 0.159 | 0.185 |
| Aspen | 1.610 | 1.610 | 2.243 | 1.610 |
| Balsam fir | 0.001 | 0.001 | 0.001 | 0.001 |
| Black spruce | 0.283 | 0.282 | 0.210 | 0.282 |
| Cottonwood | 0.012 | 0.012 | 0.009 | 0.012 |
| Cypress | 0.060 | 0.059 | 0.035 | 0.060 |
| Douglas-fir | 2.310 | 2.299 | 8.351 | 2.272 |
| Engelmann spruce | 1.144 | 1.187 | 0.578 | 1.188 |
| Hemlock | 0.001 | 0.001 | 0.000 | 0.001 |
| Lodgepole pine | 7.124 | 7.180 | 4.313 | 7.222 |
| Maple | 0.002 | 0.002 | 0.002 | 0.002 |
| Mountain hemlock | 0.077 | 0.077 | 0.054 | 0.077 |
| Paper birch | 0.127 | 0.127 | 0.126 | 0.127 |
| Ponderosa pine | 0.027 | 0.026 | 0.020 | 0.026 |
| Poplar | 0.091 | 0.091 | 0.090 | 0.091 |
| Red alder | 0.026 | 0.025 | 0.050 | 0.026 |
| Redcedar | 0.920 | 0.935 | 0.421 | 0.920 |
| Sitka spruce | 0.035 | 0.034 | 0.034 | 0.035 |
| Softwoods | 0.001 | 0.001 | 0.000 | 0.001 |
| Spruce | 2.987 | 2.871 | 2.173 | 2.849 |
| Subalpine fir | 1.862 | 1.824 | 1.293 | 1.823 |
| Tamarack | 0.001 | 0.001 | 0.001 | 0.001 |
| Western hemlock | 1.278 | 1.234 | 1.047 | 1.243 |
| Western larch | 0.174 | 0.171 | 0.183 | 0.166 |
| Western white pine | 0.003 | 0.003 | 0.003 | 0.003 |
| White spruce | 1.862 | 1.971 | 0.828 | 1.978 |
| Whitebark pine | 0.005 | 0.005 | 0.004 | 0.005 |
| Willow | 0.000 | 0.000 | 0.000 | 0.000 |

**Figure S2.** Annual net change in GHG component emissions for the *Fire Tolerant* scenario relative to the *Baseline* scenario, for five individual draws. Draws were selected from 100 Monte Carlo runs according to the 10^th^, 25^th^, 50^th^, 75^th^, 90^th^ percentiles of cumulative area burned. A negative sign indicates a smaller source of emissions or greater removals, relative to the *Baseline* scenario.
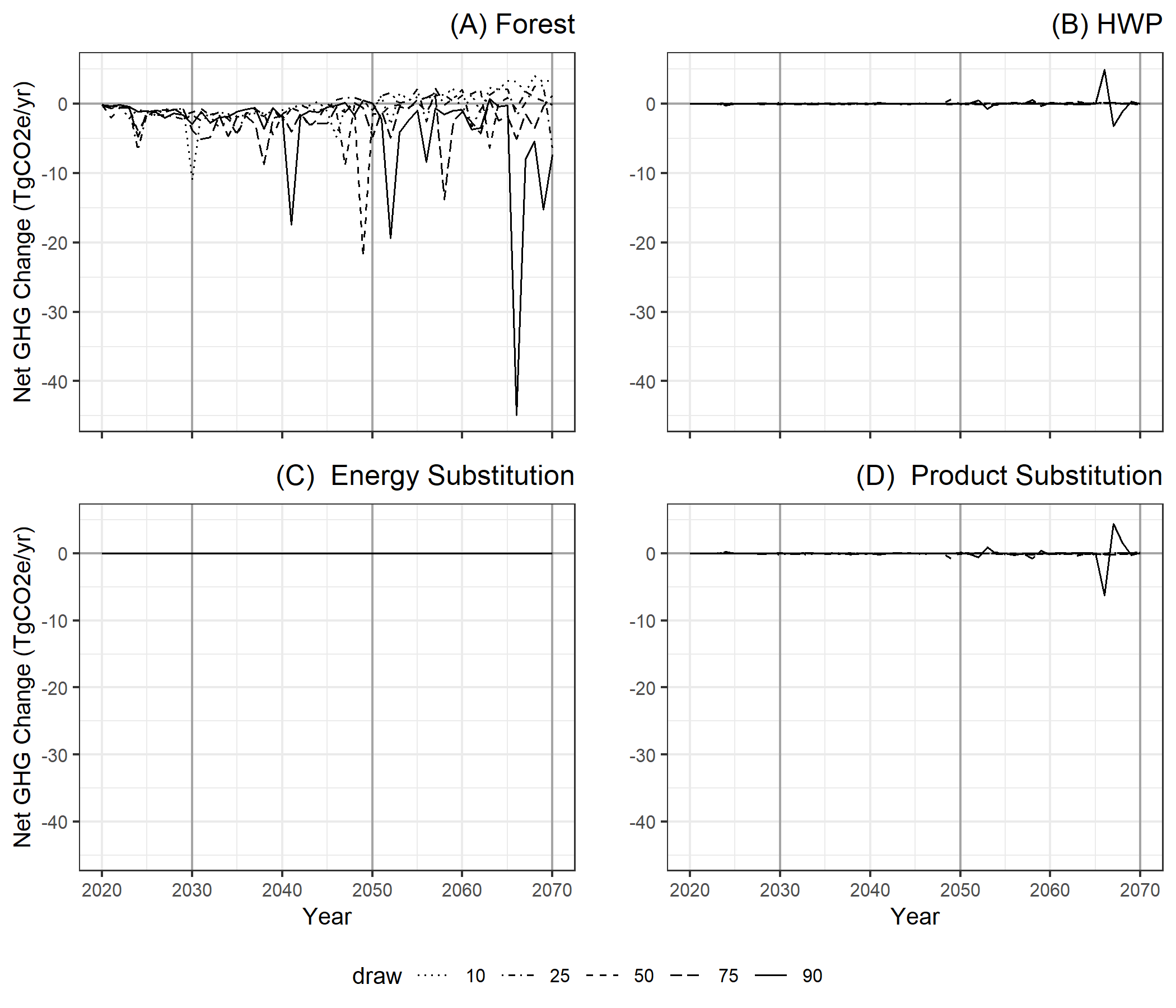


**Figure S3.** Annual net change in GHG component emissions for the *Timber* scenario relative to the *Baseline* scenario, for five individual draws.


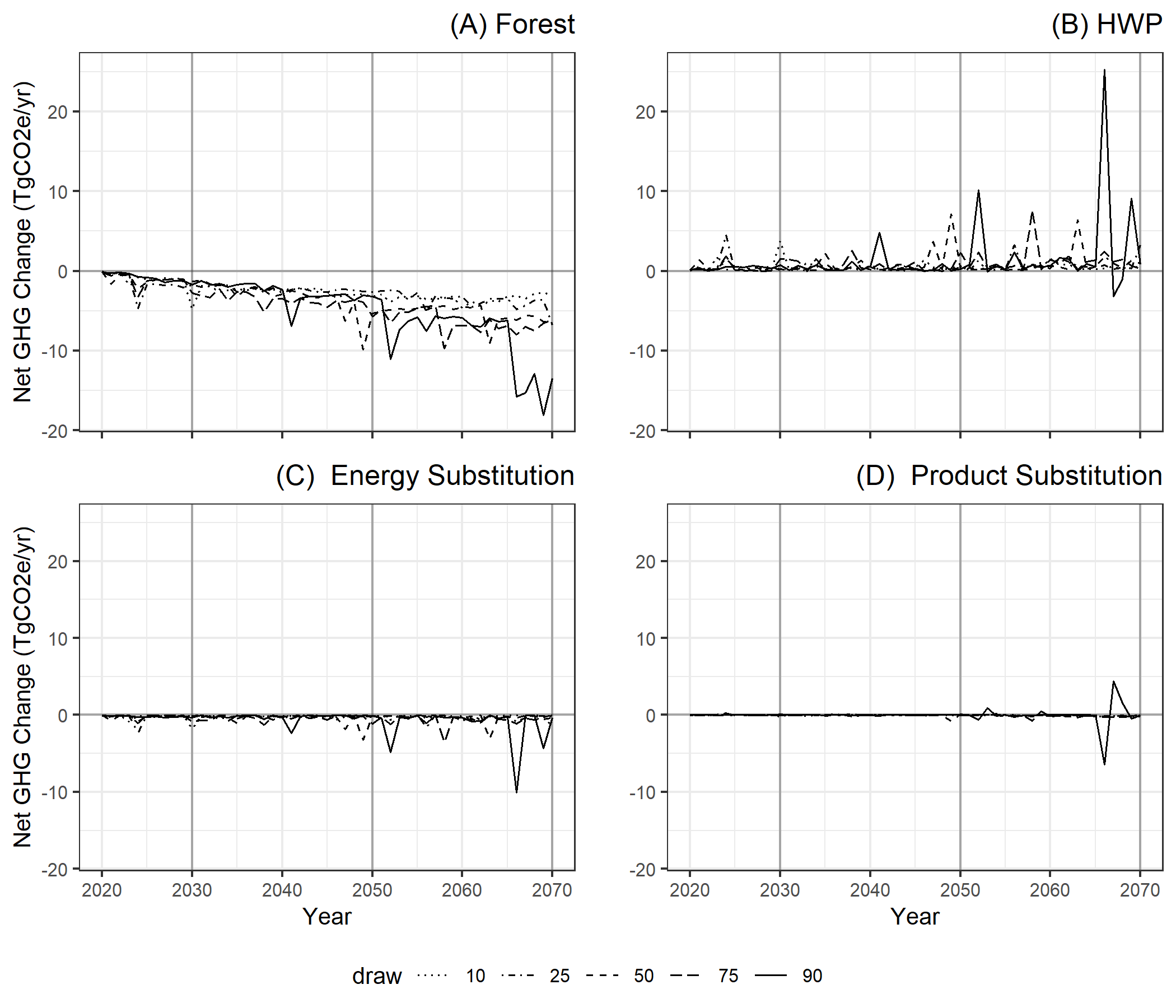


**Figure S4.** Annual net change in GHG component emissions for the *Conservation* scenario relative to the *Baseline* scenario, for five individual draws.


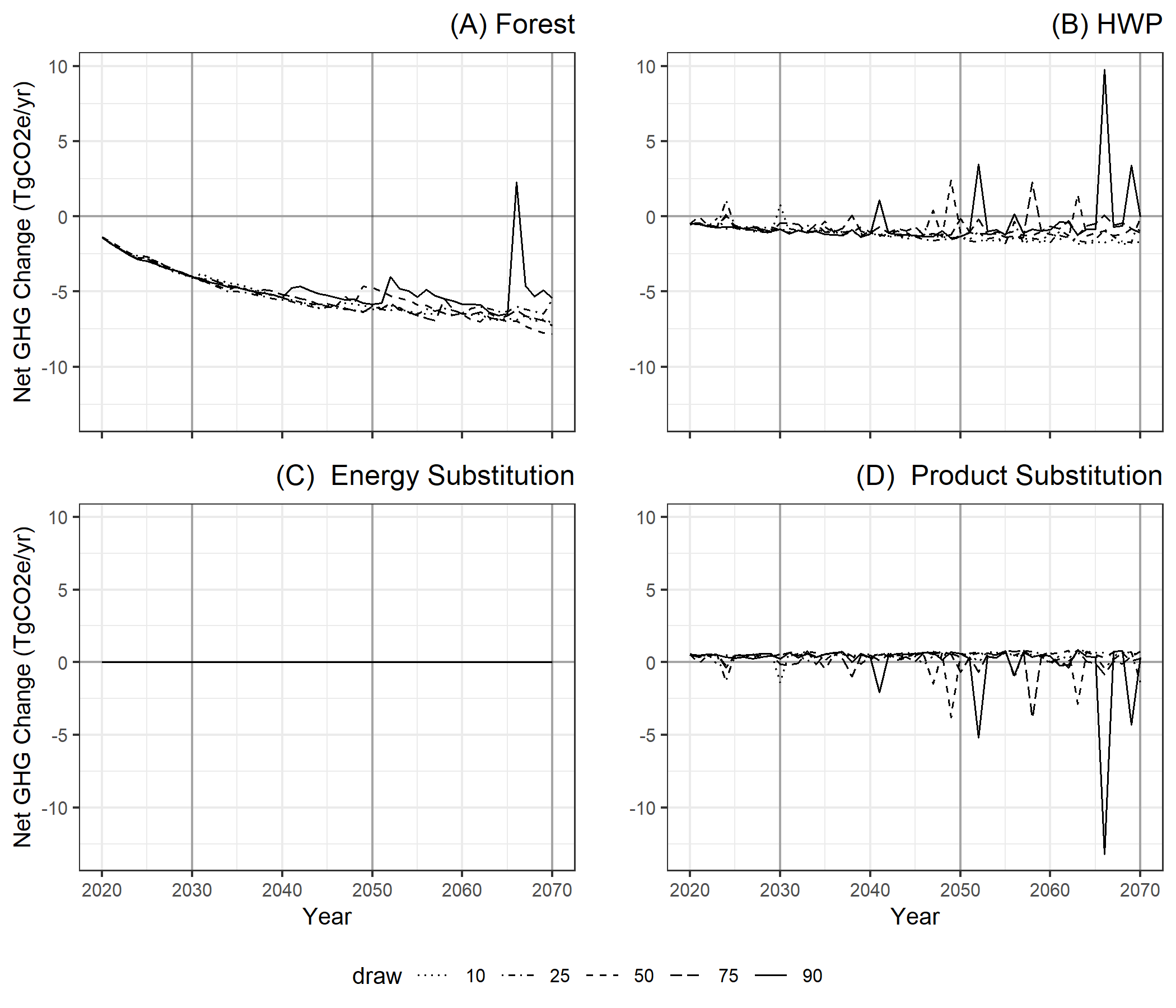


# **Supplemental File 1** spreadsheet of disturbance matrix coefficients.

# **Supplemental File 2** disturbance controller description.
